# Existing and emerging therapies for the treatment of familial hypercholesterolemia

**DOI:** 10.1016/j.jlr.2021.100060

**Published:** 2021-03-12

**Authors:** Robert S. Rosenson

**Affiliations:** Zena and Michael A. Wiener Cardiovascular Institute, Marie-Josee and Henry R. Kravis Center for Cardiovascular Health. Mount Sinai Heart, Icahn School of Medicine at Mount Sinai, New York, NY, USA

**Keywords:** angiopoietin-like protein 3, familial hypercholesterolemia, LDL, LDL receptor, lipoprotein (a), proprotein convertase subtilisin Kexin 9, cholesterol-lowering therapies, atherosclerotic cardiovascular disease, genetics, ANGPLT3, angiopoietin-like protein 3, apo B, apolipoprotein B, ASCVD, atherosclerotic cardiovascular disease, CI, confidence interval, FH, familial hypercholesterolemia, HeFH, heterozygous FH, HoFH, homozygous FH, LDLR, LDL receptor, LDLRAP1, low-density lipoprotein receptor adaptor protein 1, Lp(a), lipoprotein a, PCSK9, proprotein convertase subtilisin Kexin type 9

## Abstract

Familial hypercholesterolemia (FH), an autosomal dominant disorder of LDL metabolism that is characterized by elevated LDL-cholesterol, is commonly encountered in patients with atherosclerotic coronary heart disease. Combinations of cholesterol-lowering therapies are often used to lower LDL-cholesterol in patients with FH; however, current treatment goals for LDL-cholesterol are rarely achieved in patients with homozygous FH (HoFH) and are difficult to achieve in patients with heterozygous FH (HeFH). Therapies that lower LDL-cholesterol through LDL receptor-mediated mechanisms have thus far been largely ineffective in patients with HoFH, particularly in those with negligible (<2%) LDL receptor activity. Among patients with HeFH who were at very high risk for atherosclerotic cardiovascular disease events, combined therapy consisting of a high dose of high-intensity statin, ezetimibe, and proprotein convertase subtilisin Kexin type 9 inhibitor failed to lower LDL-cholesterol to minimal acceptable goals in more than 50%. This article provides a framework for the use of available and emerging treatments that lower LDL-cholesterol in adult patients with HoFH and HeFH. A framework is provided for the use of angiopoietin-like protein 3 inhibitors in the treatment of HoFH and HeFH.

Familial hypercholesterolemia (FH) is an autosomal dominant genetic disorder characterized by lifelong elevations in LDL-cholesterol (1). Because LDL is a causal and increscent risk factor for atherosclerotic cardiovascular disease (ASCVD) (2), individuals with FH have a high risk of early onset coronary heart disease (3, 4, 5). FH-specific guidelines recommend early detection of children and adults with FH in order to initiate LDL-cholesterol-lowering therapy and prevent premature ASCVD and death (www.nice.org.uk/guidance/cg71) (6, 7, 8, 9, 10). Optimal care of patients with FH requires a model of care based on an integrated evidence-based system of health care services (11). Fundamental components of this framework comprise screening and detection, clinical and genetic testing, assessment of risk, and implementation of lifestyle, pharmacotherapies, and apheresis procedures. Effective care requires a patient-centered approach that encompasses organization of clinical services and education through regular outpatient visits emphasizing adherence to a healthy lifestyle and pharmacotherapy, treatment of nonlipid risk factors, and community support from patient networks (8, 11).

This review discusses the use of existing cholesterol-lowering therapies and the challenges in achieving minimal acceptable LDL-cholesterol goals with available therapies in adults with homozygous FH (HoFH) and heterozygous FH (HeFH). Special emphasis is devoted to the biology, genetics, and clinical studies with inhibitors of angiopoietin-like protein 3 (ANGPLT3).

## Genetics of FH

FH is an autosomal dominant inherited disorder that results in elevations in LDL-cholesterol and increased risk for coronary heart disease (1). FH is caused mainly by mutations in *LDLR*, the gene encoding the LDL receptor (LDLR), and less often by mutations in *APOB*, the ligand for LDLR and proprotein convertase subtilisin Kexin type 9 (*PCSK9*), a protein that degrades LDLR (12). The variant frequency varies by race and ethnicity and the presence of founder genes. HoFH is characterized by two mutations in the same gene (true homozygotes) or two different genes (compound heterozygotes) (7), whereas HeFH results from a mutation in one gene (13). FH is associated with variable LDL-cholesterol levels that depend on the genetic variants contributing to the LDLR activity (14). The highest LDL-cholesterol levels occur in patients with virtually absent (null) or impaired (non-null) LDLR activity. LDL polygenes contribute to variable LDL-cholesterol levels in FH (15). In an analysis of 2,081 patients with early onset myocardial infarction (55 years and younger), a high versus low polygenic score was associated with higher untreated LDL-cholesterol levels (235 vs. 202 mg/dl) in patients with an FH mutation.

## Prevalence of FH

HoFH is a rare disorder characterized by total cholesterol levels >500 mg/dl, physical findings in the cornea (corneal arcus), skin (xanthelasmas), and tendons (xanthomata), and extremely high risk of atherosclerotic vascular disease resulting in myocardial infarction and supravalvular aortic stenosis (7). The prevalence of HoFH estimated to be 1 in 160,000 to 1 in 300,000 (1, 7, 14). In contrast, HeFH is the most common autosomal monogenic disorder affecting 30 million people in different world regions (4, 5). In comprehensive assessments of systematic reviews using genetic and validated criteria (Dutch Lipid Clinic Network, Simon Broome, World Health Organization, and region-specific diagnostic criteria), the overall prevalence of HeFH in data obtained from the most recent worldwide meta-analyses suggests that approximately 1 in 311 to 1 in 313 individuals have a clinical and/or genetically confirmed diagnosis of FH (4, 5). HeFH is encountered more commonly with higher levels of LDL-cholesterol and in patients with ischemic heart disease (4). The prevalence of HeFH is 7.2% or 23-fold higher among persons with severe hypercholesterolemia defined as an LDL-cholesterol concentration ≥190 mg/dl. Among patients with ischemic heart disease, the prevalence of HeFH is 10-fold higher than the general population, and this disorder is even more commonly encountered in patients with premature ischemic heart with a prevalence that is 20-fold higher than the general population. The LDL-cholesterol levels in patients with a severe FH phenotype often overlap with the ranges observed in patients with HoFH. When the parental history and untreated LDL-cholesterol levels are unavailable, genetic testing is often useful (11).

High lipoprotein a [Lp(a)] levels are common among patients with FH (16, 17, 18). In the Spanish Familial Hypercholesterolemia Cohort Study that included 755 index cases of HeFH and 2,927 family members, elevated Lp(a) levels defined as ≥50 mg/dl were detected in 29.6% of patients with genetically confirmed FH (19). Thus, screening for Lp(a) in HeFH identified 1 in 2.4 individuals with high Lp(a). In the British Columbia Familial Hypercholesterolemia cohort, 35.8% had Lp(a) levels ≥50 mg/dl (18). High Lp(a) levels in individuals with HeFH are associated with a higher risk of premature ASCVD events (19). When compared with unaffected family members, the hazard ratio for ASCVD events was 2.47 for individuals with HeFH and 4.40 for those with both HeFH and an elevated Lp(a).

## Guidelines for cardiovascular risk reduction in FH

Consensus statements on the management of FH from other professional societies recognize the need for early detection and intensive treatment for both children and adults with FH (www.nice.org.uk/guidance/cg71) (6, 7, 8, 9, 10, 20, 21, 22, 23). The absence of specific clinical trial data of cardiovascular disease outcomes in FH precludes strict therapeutic targets for LDL-cholesterol. In general, national and international position statements for treatment of FH in adults include the use of diet and pharmacotherapy to achieve ≥50% reduction in LDL-cholesterol from baseline and an LDL-cholesterol level <70 mg/dl or <100 mg/dl for primary prevention and <55 mg/dl or <70 mg/dl for secondary prevention. These consensus documents conform with standards of practice established in formal guideline recommendations for cholesterol (11, 12). The American Heart Association/American College of Cardiology multispecialty society guidelines recommend the use of high-intensity statin to lower LDL-cholesterol by a minimum of 50% in high-risk and very high-risk patients, whereas very high-risk patients are recommended to achieve a minimal acceptable LDL-cholesterol goal <70 mg/dl (24). The European Society of Cardiology/European Atherosclerosis Society guidelines for the management of dyslipidemias recommend treatment to achieve a ≥50% reduction in LDL-cholesterol from baseline and an LDL-cholesterol goal of <55 mg/dl in all HeFH patients for very high-risk patients, <70 mg/dl for high-risk patients, and <100 mg/dl for other patients with HeFH (25). These aggressive targets are warranted based on the emerging compendium of data supporting lower levels of LDL-cholesterol that are particularly appropriate for patients with genetically determined lifelong hypercholesterolemia. Children with FH are counseled on diet therapy and treated with a statin beginning at age 8–10 years. The goal of therapy is an LDL-cholesterol <135 mg/dl at >10 years of age.

## Diet therapy and LDL-cholesterol lowering in FH

Lifestyle modification is the foundation for risk factor modification and maintenance of health for all individuals (26, 27). Although dietary therapy is recommended for cholesterol lowering in children and adults with FH, the available evidence supporting significant reductions in LDL-cholesterol are limited (28, 29). However, the paucity of high-quality studies should not dissuade the health care professional from counseling the patient with FH and their family at each visit on the long-term established benefits of diet therapy. Lowering of LDL-cholesterol in non-FH patients may be achieved with several diets including a Mediterranean-style or low-fat vegan diet (30). Larger reductions in LDL-cholesterol and body weight were achieved with a low-fat vegan diet versus Mediterranean-style diet of overweight adults. Consumption of plant stanols or sterols has been accompanied by incremental reductions in LDL-cholesterol of 17–22 mg/dl as reported from a meta-analysis (28).

## Available therapies for LDL-cholesterol lowering in FH

**Fig. 1:**
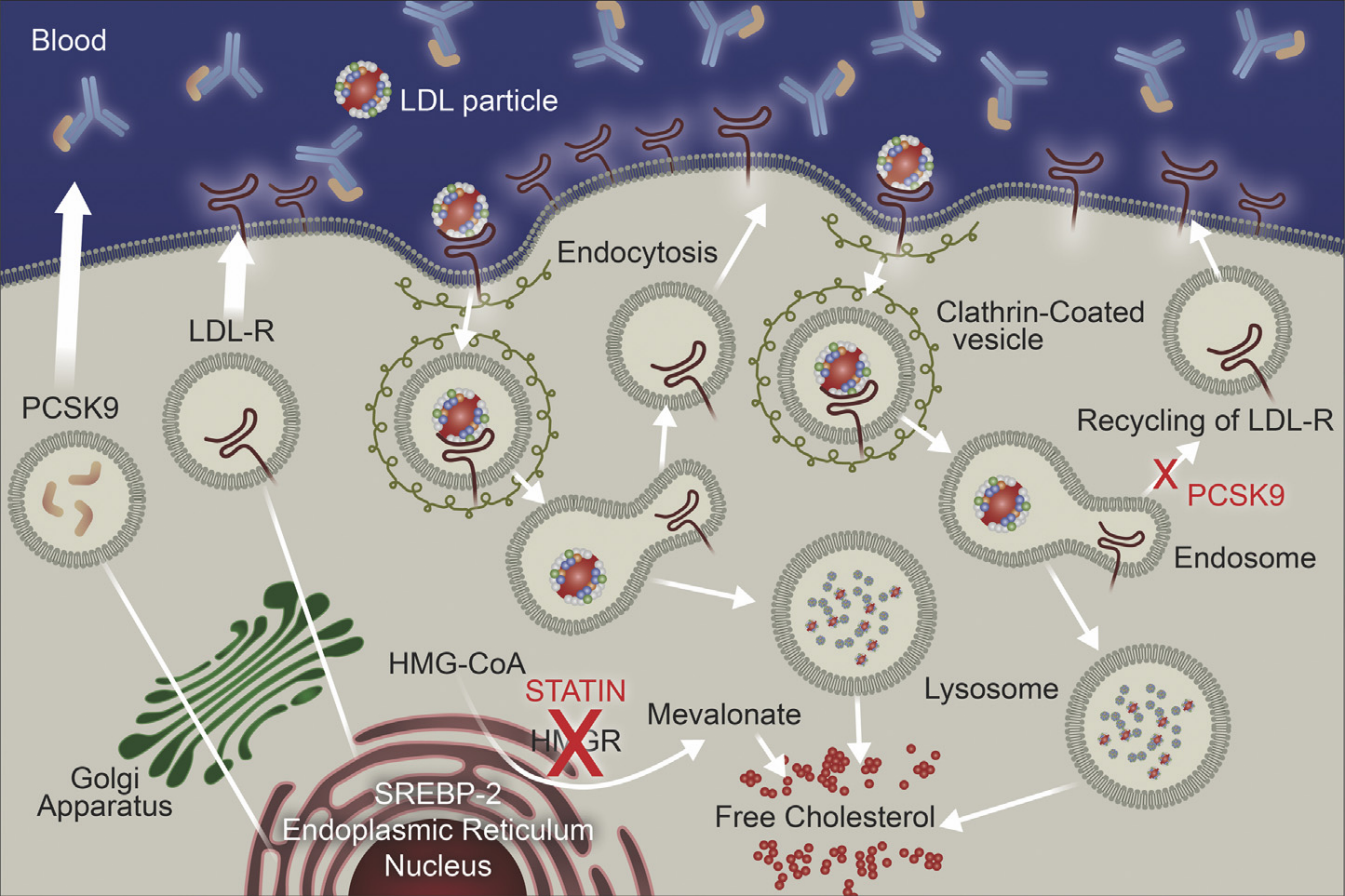
Mechanism of action for LDL lowering with statins and proprotein convertase subtilisin Kexin type 9 (PCSK9) inhibitors. The lower left of the image shows that statins inhibit HMG-CoA reductase (HMGR), which is the rate limiting step in cholesterol biosynthesis. The top section shows binding of circulating PCSK9 by human monoclonal antibody inhibitors of PCSK9. After maturation, the LDLR anchors at the cell surface and binds LDL particles via apolipoprotein B. The ligand-receptor interaction initiates receptor-mediated endocytosis. The increased pH in the lysosome degrades LDL and releases the LDLR allowing the receptor to recycle dozens of times when PCSK9 is absent. However, when PCSK9 is present, the receptor is retained in the lysosome with LDL and cannot recycle (middle right of image). Decreased intracellular cholesterol level activates SREBP-2 in the endoplasmic reticulum (lower left of image), which initiates synthesis of LDLRs allowing for binding of circulating LDL, and PCSK9 synthesis. 1. Top of form. 2. Bottom of form.

Statins represent first-line therapy for lowering LDL-cholesterol in adult patients with FH (24, 25). These recommendations are based on extensive clinical trial evidence in patients at risk for a cardiovascular event and in patients with ASCVD (31). These agents inhibit HMG-CoA, the rate-limiting step in cholesterol biosynthesis, and consequently increase LDLR activity (Fig. 1). The safety and efficacy of statins has been established in children with FH, and this therapy is considered a mainstay of treatment (32). Ezetimibe is the most commonly recommended second-line therapy for lowering LDL-cholesterol (24, 25). This therapy inhibits cholesterol transport in the jejunum by blocking the Niemann-Pick C1-like protein and reduce cholesterol availability to the liver. The use of conventional cholesterol-lowering therapy (statins and statin plus ezetimibe) was associated with improved survival in a study of 149 patients from South Africa with HoFH (33). The Cox proportional hazard ratio for mortality was 0.34 [95% confidence interval (95% CI), 0.14–0.86; *P* = 0.02]. This benefit occurred despite a modest lowering in LDL-cholesterol of 26.4% (33).

This figure was provided by Regeneron Pharmaceuticals.

## Bempedoic acid

Bempedoic acid is an inhibitor of acyl citrate lyase that has been evaluated predominantly in patients who have statin intolerance. A phase 3A trial investigated the safety and efficacy of bempedoic acid in patients with atherosclerotic vascular disease (n = 735) or HeFH (n = 45) with LDL-cholesterol levels ≥70 mg/dl on treatment with maximally tolerated lipid-lowering therapy. Treatment with bempedoic acid (180 mg daily) lowered LDL-cholesterol (15.1%) versus +2.4% with placebo (difference: −17.4% [95% CI: −21.0 to −13.9; *P* < 0.001]) (34). Bempedoic acid provides modest but incremental LDL-cholesterol lowering that may allow some patients with FH to achieve minimal acceptable LDL-cholesterol goals that are not attained with other therapies with proven cardiovascular benefits.

Other therapies that are uncommonly used for lowering LDL-cholesterol include bile acid sequestrants and nicotinic acid. The use of conventional LDL-cholesterol-lowering agents in patients with FH has been extensively reviewed by multiple professional societies (6, 7, 12, 13, 21, 22, 23, 24, 25).

## Inhibitors of PCSK9 in HeFH

**Fig. 2:**
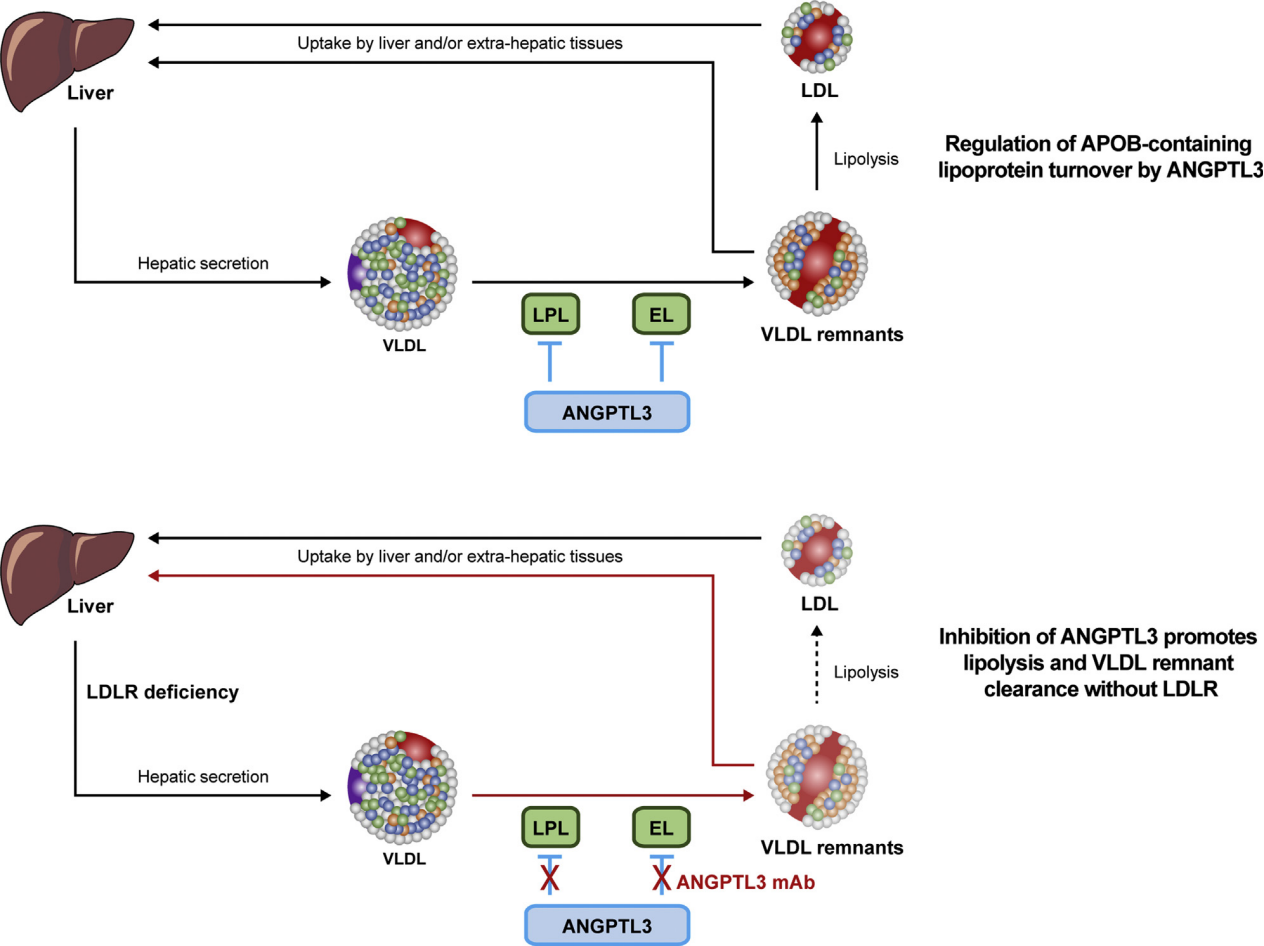
Angiopoietin-like protein 3 (ANGPTL3) inhibition lowers LDL-cholesterol. Schematic depicting the mechanism whereby ANGPTL3 inhibition lowers LDL-cholesterol. Upper panel: During homeostasis, ANGPTL3 diminishes the activity of vascular lipases lipoprotein lipase (LPL) and endothelial lipase (EL) and regulates APOB-containing lipoprotein turnover. Lower panel: Evinacumab unblocks the inhibitory effect of ANGPLT3 on both lipases, promoting VLDL remodeling and preferential removal of VLDL remnants from the circulation by hepatic remnant receptors, and reducing the pool for LDL. Thus, ANGPTL3 inhibition with a fully human monoclonal antibody lowers LDL-cholesterol by diminishing vascular LDL production. The effect of evinacumab on Apo B secretion require further study. APOB, apolipoprotein B.

LDL particles interact with hepatic LDLRs and are internalized by endosomes. Lysosomal degradation of the endosome releases the cholesterol bound to the LDLR allowing for the LDLR to return to the hepatic surface. In contrast, LDL particles that bind PCSK9 are targeted for lysosomal degradation and destruction (Fig. 2) (35, 36). Loss-of-function mutations in PCSK9 are associated with lower LDL-cholesterol levels and lifetime risk of myocardial infarction in whites and blacks and stroke in blacks (37). Clinical trials with two fully human monoclonal antibodies directed against PCSK9 have shown marked reductions in LDL-cholesterol levels and other atherogenic lipids and lipoproteins inclusive of Lp(a). Inhibitors of PCSK9 have proven benefits in the prevention of atherosclerotic cardiovascular events (35, 36, 38).

The efficacy and safety of immunotherapy for PCSK9 have encompassed multiple, international, and multicenter trials of patients with HoFH and HeFH. These trials have been conducted in adults with and without ASCVD (Table 1). A recent trial with PCSK9 inhibition in children is included as many young patients receive counseling and guidance with their parents. Alirocumab has been evaluated in multiple trials of patients with HeFH. ODYSSEY (Evaluation of Cardiovascular Outcomes After an Acute Coronary Syndrome During Treatment With Alirocumab) FH 1 and FH II were two randomized double-blinded studies that examined the safety and efficacy of alirocumab in patients with inadequate LDL-cholesterol control on maximally tolerated lipid-lowering therapy (39). LDL-cholesterol levels <70 mg/dl in FH I and FH II were achieved at week 24 by 59.8% of alirocumab-treated patients in FH I and 68.2% in FH II, respectively. The ODYSSEY HIGH FH study evaluated alirocumab versus placebo in 107 patients with HeFH and LDL-cholesterol ≥160 mg/dl on maximum tolerated statins ± other lipid-lowering therapies (40). At baseline, all patients were treated with a statin including a high-intensity statin in 73.4% of the alirocumab group and 71.4% of the placebo group. The proportion of very high cardiovascular disease risk patients achieving LDL-cholesterol levels was <70 mg/dl or high-risk patients reaching LDL-cholesterol was <100 mg/dl with 41.0% in the alirocumab group and 5.7% in the placebo group. ODYSSEY LONG TERM evaluated the efficacy of two doses of alirocumab (75 mg subcutaneous every 2 weeks and 150 mg subcutaneous every 2 weeks) on LDL-cholesterol from baseline to 12 weeks (41). Treatment with alirocumab 75 mg every 2 weeks reduced LDL-cholesterol by −47.3% from a baseline of 166.6 mg/dl. ODYSSEY OLE was an open-label extension of patients with HeFH who had completed one of four phase 3 double-blinded clinical trials (FH I, FH II, HIGH FH, and LONG TERM) (42). The dose of alirocumab either remained at 75 mg every 2 weeks or titrated to 150 mg every 2 weeks based on the physician's clinical judgment. At week 96, the average reduction in LDL-cholesterol was −46.8% for patients without a dose increase from 75 mg every 2 weeks and −55.4% in patients with a dose increase in alirocumab to 150 mg every 2 weeks.

**Table 1:** Inhibitors of PCSK9i in HeFH and HoFH patients

Trial	Study Population	Sample Size	Therapy	Design	Baseline Characteristics of Heterozygous/Homozygous Patients	Major Outcome
Inhibitors of PCSK9i in HeFH patients						
ODYSSEY FH I and FH II (39)	Adults with HeFH (39.9% genotyped, 59.8% clinical criteria in alirocumab group) LDL-C level of 70 mg/dl (1.8 mmol/l)	FH I (n = 486) FH II (n = 249)	Alirocumab	Randomized 2:1 alirocumab 75 mg SC or matching placebo every 2 weeks, increased to 150 mg every 2 weeks if LDL-C ≥70 mg/dl	• n = 323 • Mean age: 52.1 ± 12.9 years • CHD: 45.5% • CHD risk equivalents: 16.7% • Statin use: 100% • High-dose statin: 82.7% • Ezetimibe use: 56.0%	FH1: Mean placebo-corrected change in LDL-C was −57.9% from baseline (144.7 mg/dl) to week 24 (144.7 mg/dl) FH2 change in LDL-C: −51.4 from baseline (134.6 mg/dl) to 67.7 mg/dl
ODYSSEY HIGH FH (40)	HeFH and LDL-C ≥160 mg/dl on maximum tolerated statins ± other lipid-lowering therapies Adults with HeFH (70.1% genotyped, 29.9% clinical criteria in alirocumab)	n = 249	Alirocumab	Randomized 2:1 alirocumab 150 mg SC or placebo every 2 weeks	• n = 167 • Mean age: 53.2 ± 12.9 years • CHD: 34.7% • CHD risk equivalents: 9.0% • Statin use: 100% • High-dose statin: 100% • Ezetimibe use: 67.1%	Baseline LDL-C 196.3 mg/dl (in placebo, 201.0 mg/dl) Mean reductions in LDL-C from baseline to week 24 were −45.7% in the alirocumab group and −6.6% in the placebo group (placebo-corrected difference of −39.1%; *P* < 0.0001) Proportion of very high CVD risk patients achieving LDL-C levels <70 mg/dl or high-risk patients reaching LDL-C <100 mg/dl was 41.0% in alirocumab (5.7% in placebo)
ODYSSEY LONG TERM (41)	At the beginning of the open-label extension, 71.5% of patients were treated with maximally tolerated statin and 54.7% were receiving a high-intensity statin and ezetimibe	n = 107	Alirocumab	Randomized 2:1 to 2 doses of alirocumab (75 mg SC every 2 weeks and 150 mg SC every 2 weeks)	• n = 72 • Mean age: 49.8 ± 14.2 years • CHD: 43.1% • CHD risk equivalents: 18.1% • Statin use: 100% • High-dose statin: 73.6% • Ezetimibe use: 19.4%	Treatment with alirocumab 75 mg SC every 2 weeks reduced LDL-C by -47.3% from baseline 166.6 mg/dl
RUTHERFORD-2 (43)	Clinical HeFH diagnosis according to Simon Broome criteria and on stable dose of statin with or without other lipid-modifying therapy for at least 4 weeks	n = 331	Evolocumab	Randomly assigned 2:2:1:1 SC evolocumab 140 mg every 2 weeks (n = 111), evolocumab 420 mg monthly (n = 110), or SC placebo every 2 weeks (n = 55) or monthly (n = 55), for 12 weeks	• n = 110 (140 mg) • Mean age: 52.6 ± 12.3 years • CHD: 35% • Statin use: 100% • High-dose statin: 100% • LDL-C at baseline: 162.41 mg/dl • n = 110 (420 mg) • Mean age: 51.9 ± 12.0 years • CHD: 35% • Statin use: 100% • LDL-C at baseline: 154.7 mg/dl	Evolocumab therapy lowered mean LDL-C at week 12 (every-2-weeks dose: 59.2% reductions, monthly dose 61.3% reduction) Evolocumab lowered LDL-C at the mean of weeks 10 and 12 (60.2% reduction and 65.6% reduction)
TAUSSIG (44)	Severe HeFH based on suboptimal response to cholesterol-lowering therapy and the presence of CVD or risk factors	n = 194	Evolocumab	SC evolocumab 420 mg monthly or 420 mg every 2 weeks if on apheresis	• n = 194 • Mean age: 54.7 ± 11.9 years • CHD: 59.8% • Statin use: 86.1% • High-dose statin: 57.7% • Ezetimibe use: 62.4% • LDL-C at baseline: 192.7 ± 64.6 mg/dl	Larger reductions in LDL-C of −54.9 ± 17.4, −56.9 ± 19.2, and −47.2 ± 27.9% in severe HeFH than HoFH at weeks 12, 48, and 216
HAUSER-RCT (45)	Pediatric patients with HeFH; Aged 10–17 years, received stable lipid-lowering treatment for at least 4 weeks, screening LDL-C level ≥130 mg/dl Genetic: LDLR mutation (67%), apo B mutation (2%), and clinical criteria 31%	n = 157	Evolocumab	Randomly assigned 2:1 SC evolocumab 420 mg or placebo	• n = 104 • Mean age: 13.7 ± 2.3 years • CHD: 59.8% • Statin use: 100% High-intensity statin: 18% • Ezetimibe use: 12% • LDL-C: 185.0 ± 45.0 mg/dl, TC 247.3 ± 49.5 mg/dl	At week 24, mean percent change from baseline in LDL-C was −44.5% (evolocumab) vs. −6.2% (placebo), difference of −38.3%
Inhibitors of PCSK9 in HoFH						
ODYSSEY HoFH (47)	HoFH patients	n = 69	Alirocumab	Randomized 2:1 alirocumab 150 mg SC every 2 weeks or placebo	Background LLT: • Statins: 97.1% • High-intensity statins: 85.5% • Ezetimibe use: 89.6% • Lomitapide: 14.5% • Apheresis: 14.5% Treatment group: • n = 23 • TC: 429.2 mg/dl • LDL-C: 335.6 mg/dl	LDL-C was reduced from baseline by −35.6% (−26.9% in the alirocumab group vs. 8.6% in the placebo group). Lowering of LDL-C was highly variable and genotypically determined. Among the 45 patients homozygous for either LDLR, PCSK9, or LDLRAP1, treatment with alirocumab ineffective in lowering LDL-C in one-third. Reduction in Lp(a) of −28.4%
TESLA (48)	No apheresis Genotype: LDLR mutations (95%), true homozygous (45%), compound heterozygous (48%), heterozygous (3%), autosomal recessive hypercholesterolemia (3%)	n = 50	Evolocumab	Evolocumab 420 mg every 4 weeks	• n = 33 • Mean age: 30 ± 12 • CHD: 46% • Use of statins: 100% • Ezetimibe use: 91% • LDL-C ultracentrifugation: 355.8 ± 135.4 mg/dl • LDL-C calculated: 355.8 ± 135.4 mg/dl	LDL-C reduced by average 31% as compared with placebo
TAUSSIG (49)	HoFH with suboptimal LDL-C response and inadequately lowered free PCSK9 level (≥100 ng/ml) Background: stable LDL-C lowering therapy for at least 4 weeks; evolocumab 420 mg monthly in all or biweekly if on apheresis	n = 106	Evolocumab	Either evolocumab 420 mg SC every month or every 2 weeks	Background LLT: • Statins: 90.6% • High-intensity statins: 90.6% • Ezetimibe use: 89.6% • Apheresis: 32.1%	LDL-C at weeks 12, 48, and 216 reduced by −21.2 ± 25.0% Among 34 patients with HoFH undergoing apheresis, 26% were able to discontinue apheresis

CHD, coronary heart disease; HAUSER-RCT, evolocumab in pediatric heterozygous familial hypercholesterolemia-randomized clinical trial; SC, subcutaneously; TAUSSIG, Trial Assessing Long Term Use of PCSK9 Inhibition in Subjects with Genetic LDL Disorders; TC, total cholesterol; TESLA, Trial Evaluating PCSK9 Antibody in Subjects With LDL Receptor Abnormalities.

Several trials have investigated the safety and efficacy of evolocumab in patients with HeFH. RUTHERFORD-2 was randomly assigned in a 2:2:1:1 ratio to receive subcutaneous evolocumab 140 mg every 2 weeks (n = 111), evolocumab 420 mg monthly (n = 110), or subcutaneous placebo every 2 weeks (n = 55) or monthly (n = 55) for a duration of 12 weeks (43). Evolocumab therapy when compared with placebo lowered mean LDL-cholesterol at week 12 (every-2-weeks dose: −59.2% reduction; monthly dose: −61.3% reduction; and −60.2% reduction at the mean of weeks 10 and 12). The TAUSSIG study included 194 patients with severe heterozygous FH as determined by the investigator based on suboptimal response to cholesterol lowering therapy and the presence of atherosclerotic cardiovascular disease or other cardiovascular disease risk factors. (44) Larger reductions in LDL cholesterol were observed in severe heterozygous FH patients than homozygous FH patients at weeks 12, 48 and 216. Evolocumab in pediatric heterozygous familial hypercholesterolemia-randomized clinical trial was a 24-week, randomized, double-blind, placebo-controlled trial to evaluate the efficacy and safety of evolocumab in 157 pediatric patients with heterozygous FH. (45). Eligible patients were 10–17 years of age, received stable lipid-lowering treatment for at least 4 weeks, and a screening LDL-cholesterol level of ≥130 mg/dl. Treatment with monthly subcutaneous injections of evolocumab (420 mg) or placebo reduced LDL-cholesterol level by −44.5% in the evolocumab group and −6.2% in the placebo group, for a difference of −38.3% (*P* < 0.001).

## Small interfering RNA therapy in HeFH

Inclisiran is a small interfering RNA therapy that inhibits hepatic synthesis of PCSK9 (35). Inclisiran in patients at high cardiovascular risk with elevated LDL cholesterol-9 randomly assigned 482 adults with HeFH to receive subcutaneous injections of inclisiran sodium (at a dose of 300 mg) or matching placebo on days 1, 90, 270, and 450 (46). The two primary end points were the percent change from baseline in the LDL-cholesterol level on day 510 and the time-adjusted percent change from baseline in the LDL-cholesterol level between day 90 and day 540. The mean baseline LDL-cholesterol level was 153 mg/dl. At day 510, the percent change in the LDL-cholesterol level was a reduction of −39.7% (95% CI, −43.7 to −35.7) in the inclisiran group versus an increase of 8.2% (95% CI, 4.3–12.2) in the placebo group resulting in a between-group difference in LDL-cholesterol of −47.9% (95% CI, −53.5 to −42.3; *P* < 0.001). The time-adjusted change in LDL-cholesterol level between day 90 and day 540 was −38.1% (95% CI, −41.1 to −35.1) in the inclisiran group and an increase of 6.2% (95% CI, 3.3–9.2) in the placebo group, for a between-group difference of −44.3% (95% CI, −48.5 to −40.1; *P* < 0.001).

## Inhibitors of PCSK9 in HoFH

The efficacy and safety of alirocumab in adults with HoFH was investigated in the ODYSSEY HoFH (Study in Participants With Homozygous Familial Hypercholesterolemia) (47). This study enrolled 69 patients who were randomized 2:1 to alirocumab or placebo. Background therapy included statins in 67 (59 patients on high-intensity statins), 50 patients on ezetimibe, 10 patients on lomitapide, and 10 patients undergoing apheresis. The mean baseline LDL-cholesterol level was 259.6 ± 154.6 mg/dl. In the double-blind placebo-controlled phase of the trial, treatment with alirocumab 150 mg subcutaneous administration every 2 weeks, LDL-cholesterol was reduced from baseline by −35.6% (−26.9% in the alirocumab group vs. 8.6% in the placebo group). Lowering of LDL-cholesterol was highly variable based on residual LDLR activity. Treatment with alirocumab was ineffective in lowering LDL-cholesterol in one-third of patients homozygous for either *LDLR*, *PCSK9*, or low-density lipoprotein receptor adaptor protein 1 (*LDLRAP1*). Reductions in other atherogenic lipoproteins included reductions in non-HDL cholesterol of −32.9%, apolipoprotein B (apo B) of −29.8%, and Lp(a) of −28.4%.

The Trial Evaluating PCSK9 Antibody in Subjects With LDL Receptor Abnormalities Part B study enrolled 50 patients, none receiving LDL apheresis, and reported that administration of evolocumab 420 mg every 4 weeks reduced LDL-cholesterol by an average 31% compared with placebo (48). The Trial Assessing Long Term USe of PCSK9 Inhibition in Subjects With Genetic LDL Disorders enrolled 300 patients with FH including 106 with HoFH (49). The study investigated the efficacy of evolocumab 420 mg administered subcutaneously every month or every 2 weeks. Study participants received treatment with either evolocumab 420 mg subcutaneous administration once monthly or evolocumab 420 mg every 2 weeks if the LDL-cholesterol response was considered suboptimal and the free PCSK9 level was inadequately lowered (≥100 ng/ml). In the patients with HoFH, treatment with evolocumab reduced LDL-cholesterol at weeks 12, 48, and 216 by −21.2 ± 25.0%, −24.8 ± 31.7%, and −24.0 ± 41.3%, respectively (44). Among 34 patients with HoFH undergoing apheresis, 26% were able to discontinue apheresis.

The trials conducted in patients with HoFH demonstrate statistically significant reductions in LDL-cholesterol; however, the response is highly variable and depends on the genotype. Lesser reductions in LDL-cholesterol are reported for patients with HoFH traits versus compound heterozygous traits.

## Inhibitors of microsomal transport protein in HoFH

Lomitapide is an inhibitor of the microsomal triglyceride transport protein. Microsomal triglyceride transport protein is involved in the intracellular assembly of VLDL and secretion of apo B-containing VLDL. In a single-arm, open-label, and phase 3 study of lomitapide, 29 patients with HoFH participated in a dose escalation study of lomitapide 5 mg to a maximum of 60 mg daily based on tolerability and safety (50). The primary end point was mean percent change in levels of LDL-cholesterol from baseline to week 26. After completion of the efficacy phase, patients remained on treatment with lomitapide (median dose, 40 mg daily) to week 78 for safety assessment. LDL-cholesterol fell by 50% (95% CI, −62 to −39) from a baseline mean ± SD of 336 ± 112 to 166 ± 96 mg/dl. Lesser reductions in LDL-cholesterol of −38% (−52 to −24; *P* < 0.0001) were reported at week 78. The most common adverse events were gastrointestinal symptoms. Levels of aminotransaminase >5-fold above the upper limit of normal were reported in four patients. These changes resolved with dose reduction or temporary interruption of lomitapide. A long-term extension study was conducted in the 19 of the 23 patients who completed the phase 3 open-label trial. LDL-cholesterol was lowered by 45–50% during the 294 weeks of treatment (51). There was a 12% increase in hepatic fat and an increase in transaminases >5-fold above the upper limit of normal range in 21%. All patients were counseled on a low-fat diet (<20% calories from fat) and prescribed daily intake of supplements that included vitamin E (400 IU) and fatty acids (200 mg linoleic acid, 210 mg alpha linolenic acid, 110 mg eicosopentaenoic acid, and 80 mg of docosahexaenoic acid). In a phase 3 dose-ranging study of Japanese patients with HoFH (52), the extension study enrolled five of the eight patients who completed the 56-week phase 3 dose-escalation study. The average daily dose of lomitapide was 22.0 mg (range, 10–40). At week 12, treatment with lomitapide reduced LDL-cholesterol by −31.9% from a derived baseline. A sustained reduction in LDL-cholesterol lowering was reported at week 60.

## LDL apheresis in HoFH

LDL apheresis is reserved for use in children and adults with HoFH and adults refractory HeFH who have inadequate response to pharmacological therapy (10, 53). This invasive strategy is particularly valuable in the treatment of HoFH patients with two null mutations in LDLR or no LDLR activity. A systematic review of studies published between 2000 and 2013 reported that the mean change in LDL-cholesterol after apheresis was 57–75% in patients with HoFH and 58–63% for patients with HeFH (54). Cardiovascular benefits of LDL apheresis derive from registries that report associations with the magnitude of LDL-cholesterol lowering and the cumulative exposure to LDL-cholesterol (55, 56, 57).

Limitations of LDL apheresis are manifold including access to regional centers, high costs, and patient inconvenience (54). Other concerns include depletion of apolipoprotein E-HDL and pre-beta1-HDL in the immediate postapheresis period suggesting defective cellular efflux (58) and transient activation of the complement system and inflammatory cytokine network (59). However, apheresis has other putative benefits including lowering Lp(a) levels by 64% as reported in a single-site experience of 14 patients with a mean LDL-cholesterol of 80 mg/dl (60).

## New therapies for LDL-cholesterol lowering in FH

Despite extremely high LDL-cholesterol levels and a high prevalence of ASCVD in patients with HeFH, LDL-cholesterol targets of <70 and <100 mg/dl are achieved by 22% and 48%, respectively, despite use of two or three cholesterol-lowering agents inclusive of a statin, an ezetimibe, and/or a PCSK9 inhibitor (61, 62). A real-world study of 143 patients with HeFH and very high-risk status for cardiovascular disease revealed that only 45% achieved an LDL-cholesterol level of <55 mg/dl on triple therapy including a high-intensity statin, an ezetimibe, and a PCSK9 inhibitor (63).

ANGPTL3 is an inhibitor of lipoprotein lipase and endothelial lipase and thus a key regulator of lipid metabolism. Loss-of-function mutations in ANGPTL3 are associated with lower levels of LDL-cholesterol, triglycerides, and HDL-cholesterol. In an analysis of patients with ANGPTL3 deficiency, a condition known as familial hypobetalipoproteinemia, the LDL-cholesterol levels were 67% lower in homozygotes and 9% lower in heterozygotes when compared with unaffected familial controls (64). Large genome-wide association studies have confirmed these observations. In a pooled analysis of 58,335 participants in the DisovEHR human genetics study, the 212 carriers versus the 49,017 noncarriers of loss-of-function variants in ANGPTL3 had lower levels of LDL-cholesterol levels, HDL-cholesterol, and triglycerides (65). An analysis of 18,231 participants in the Myocardial Infarction Genetics Consortium reported that loss-of-function variants in ANGPTL3 were associated with 11.8% (95% CI, −21.5 to −2.1) lower levels of LDL-cholesterol and triglycerides than controls but no difference in HDL-cholesterol (66).

Genetic inactivation of ANGPTL3 is associated with a lower risk of ischemic heart disease. The DisovEHR human genetics study reported an odds ratio of 0.60 (0.41–0.86) for coronary artery disease in carriers versus noncarriers of ANGPTL3 loss-of-function variants (51). Similar findings were reported from the Myocardial Infarction Genetics Consortium (65). In this meta-analysis of Mendelian randomization studies, carriers had an odds ratio for coronary artery disease of 0.66 (0.44–0.98) compared with noncarriers.

In hyperlipidemic humans and mice, ANGPTL3 inhibition with evinacumab mediates endothelial lipase-dependent pathway that reduces VLDL lipid content and particle size facilitating efficient clearance of remnant particles from the circulation (67). The reduction in VLDL remnants suggests that ANGPLT3 inhibition reduces LDL production resulting in lower LDL-cholesterol levels. The efficacy of evinacumab on atherosclerotic plaque lesions and lesion composition was investigated in an experimental model of hyperlipidemic mice (APOE∗3-Leiden.cholesteryl ester transfer protein mice) fed a Western-type diet (68). When compared with the group receiving diet therapy, treatment with atorvastatin reduced atherosclerosis progression by 28%. Combined treatment with atorvastatin and alirocumab completely blocked progression and decreased lesion severity, whereas triple therapy with atorvastatin, alirocumab, and evinacumab regressed lesion size from baseline in the thoracic aorta by 50% and the aortic root by 36% (*P* < 0.05 vs. baseline) and improved lesion composition as shown by reduced monocyte adherence to the endothelium and the number of proliferative macrophages in the plaque with consequent regression of plaque lesions. In *LDLr*^−/−^ mice fed a Western diet, treatment with an *Angptl3* antisense oligonucleotide delated progression of en face atherosclerosis by 52% when compared with mice in the control group (69).

## ANGPLT3 inhibition in HoFH

The efficacy and safety of evinacumab, a fully human monoclonal antibody against ANGPTL3, was investigated in the Efficacy and Safety of Evinacumab in Patients With Homozygous Familial Hypercholesterolemia trial (70). This double-blind, placebo-controlled, and phase 3 trial included 65 patients with HoFH who were receiving stable lipid-lowering therapy. Eligible participants were randomly assigned in a 2:1 ratio to receive an intravenous infusion of evinacumab (at a dose of 15 mg per kilogram of body weight) every 4 weeks or placebo. The primary outcome was the percent change from baseline in the LDL-cholesterol level at week 24 of double-blind treatment. A diagnosis of HoFH was based on at least one of the following criteria: documented functional mutation/mutations in both LDLR alleles, the presence of homozygous or compound heterozygous mutations in apo B or PCSK9, double heterozygotes or patients with homozygous LDLRAP1 mutations or an untreated total cholesterol level >500 mg/dl and triglycerides <300 mg/dl, and either both parents with documented total cholesterol >250 mg/dl or cutaneous or tendinous xanthoma before 10 years of age. The genotype status in the placebo and evinacumab groups with null-null mutations was 27.2–34.9%, respectively, including 9.1% and 18.6% with LDLR activity <2%. Non-null/null mutations were present in 72.7% of placebo-treated patients and 65.1% of evinacumab-treated patients. At baseline, most patients received combinations of lipid-lowering therapy including apheresis.

The mean baseline LDL-cholesterol level in the two groups was 255.1 mg/dl on the background of maximum tolerated doses of background lipid-lowering therapy that may have included LDL apheresis. At week 24, the between-group least-squares mean (SD) percent difference in LDL-cholesterol was –49.0% (95% CI, –65.0 to –33.1; *P* < 0.001) and the between-group least-squares mean absolute difference in the LDL-cholesterol level was –132.1 mg/dl (95% CI, –175.3 to –88.9; *P* < 0.001). The LDL-cholesterol level was lower in the evinacumab group than in the placebo group in patients with null-null variants (–43.4% vs. +16.2%) and in those with non-null variants (–49.1% vs. –3.8%). In the subgroup of patients with LDLR activity <2%, 24 weeks' treatment with evinacumab reduced LDL-cholesterol by −53.5% versus an increase of 18.8% with placebo (least square mean difference −72.3%, *P* = 0.0005) (71). This reduction in LDL-cholesterol was not different than the overall population. Overall, 53.8% of patients with HoFH had ASCVD. For this subgroup with ASCVD, 43.5% of the evinacumab group and 36.4% of the placebo group achieved LDL-cholesterol levels <100 mg/dl, 26.1% versus 9.1% achieved LDL-cholesterol levels <70 mg/dl, and 13.0% versus 0.0% achieved LDL-cholesterol level <55 mg/dl at week 24 (72). In addition to lowering LDL-cholesterol, treatment with evinacumab lowered concentrations of other atherogenic lipoproteins including apo B [−36.9% (95% CI, 48.6–25.2); *P* < 0.0001] and non-HDL cholesterol [−51.7% (64.8–38.5)]. Treatment emergent adverse events were similar in the two groups. Limitations of this study include the duration and number of patients, particularly for conclusions regarding long-term safety of evinacumab. The safety of evinacumab will be further assessed in the open-label extension phase of the trial (*ClinicalTrials.gov* identifier: NCT03409744).

**Fig. 3:**
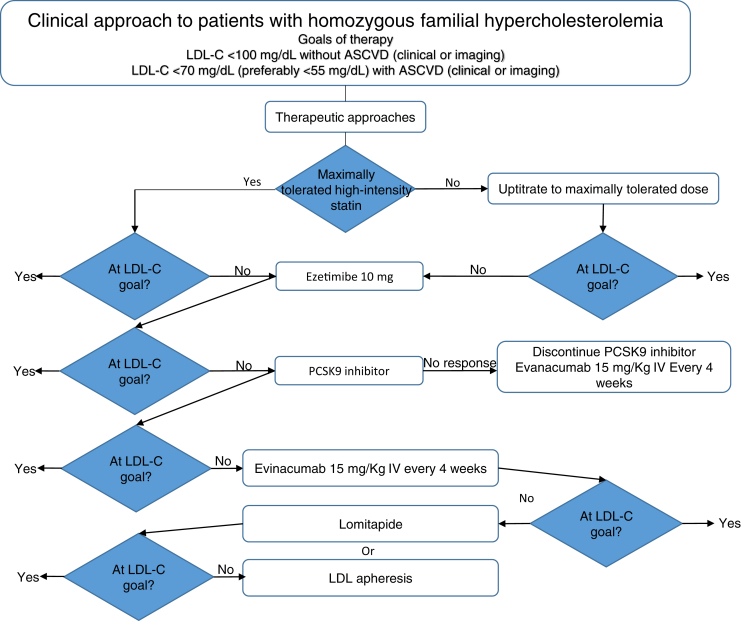
Clinical algorithm for LDL-cholesterol lowering in patients with homozygous familial hypercholesterolemia. ASCVD, atherosclerotic cardiovascular disease; PCSK9, proprotein convertase subtilisin Kexin type 9.

Evinacumab may provide an effective treatment option for patients with HoFH who are unable to reach target LDL-cholesterol despite multiple conventional lipid-lowering therapies and apheresis (Fig. 3). Among HoFH patients with null-null mutations in LDLR and no remaining LDLR activity, evinacumab may be particularly beneficial in lowering levels of LDL-cholesterol. The use of evinacumab in patients undergoing apheresis improves LDL-cholesterol control and reduces the frequency of apheresis procedures for some patients.

## ANGPLT3 inhibition in HeFH

A global multicenter trial of 272 patients with refractory hypercholesterolemia, including 72% with HeFH, investigated the efficacy of evinacumab administered either intravenously or subcutaneously. This phase 2 trial included patients with “refractory hypercholesterolemia” defined by an LDL-cholesterol level of ≥70 mg/dl or higher in the presence of ASCVD or ≥100 mg/dl in the absence of atherosclerotic cardiovascular on background treatment with maximally tolerated statins with or without ezetimibe and the highest available dose of a PCSK9 inhibitor (73). Eligible patients were randomized to receive treatment with either of two regimens of intravenous evinacumab or placebo or three regimens of subcutaneous evinacumab or placebo. The primary end point was percent change in LDL-cholesterol at week 16 with intravenous or subcutaneous evinacumab or placebo.

### Intravenous treatment

The baseline mean (±SD) of LDL-cholesterol was similar in the intravenous groups assigned to evinacumab 5 mg/kg [146.0 ± 61.0 mg/dl; evinacumab 15 mg/kg (141.1 ± 54.4 mg/dl)] and placebo (144.5 ± 46.6 mg/dl). At week 16, the least squares mean differences in LDL-cholesterol in the groups assigned to receive intravenous evinacumab (vs. placebo) at a dose of 5 and 15 mg/kg was −24.2% and −50.5%, respectively (*P* < 0.0001). Dose-dependent reductions were observed in the concentrations of other atherogenic lipoproteins (apo B, non-HDL cholesterol, and triglycerides) with the exception of Lp(a) that was reduced by −16.5% in both groups receiving evinacumab. In the intravenous group, there was one adverse event of special interest leading to discontinuation of therapy was an anaphylactic reaction that the investigator considered related to the study medication. This event occurred in a patient treated with evinacumab 15 mg/kg intravenous who had a relevant medical history of seasonal allergies and asthma included multiple symptoms including dyspnea, dizziness with associated reduced blood pressure and increased heart rate, chest pressure, and tingling in the arms and legs. This event resolved within hours after discontinuation of the infusion and treatment with oral diphenhydramine.

### Subcutaneous treatment

The baseline mean (±SD) of LDL-cholesterol was similar among patients assigned to evinacumab 300 mg every 2 weeks (136.2 ± 70.2), evinacumab 300 mg every week (159.1 ± 73.0 mg/dl), evinacumab 450 mg every week (146.3 ± 84.6 mg/dl), and placebo (157.8 ± 92.4 mg/dl). At week 16, the least squares mean difference (vs. placebo) with evinacumab 300 mg every2 weeks was −38.5% (*P* < 0.0001), evinacumab 300 mg every week of −52.9%, and evinacumab 450 mg every week (−56.0%). Dose-dependent reductions were observed in other atherogenic lipoprotein levels (apo B, non-HDL-cholesterol, and triglycerides) except for Lp(a) that was reduced in a non-dose-dependent manner. HDL-cholesterol levels were also reduced. Few serious adverse safety events were observed with subcutaneous treatment. In the evinacumab 300 mg every 2-week group, there was one case of dyspnea leading to discontinuation of therapy and dyspnea deemed related to the study drug. Limitations of the trial include the low use of ezetimibe, which ranged from 34 to 43% in the intravenous treatment groups and from 18 to 36% in the subcutaneous treatment groups. At the time of publication, there was incomplete genotyping for FH traits in participants with refractory hypercholesterolemia, and the open-label treatment period was incomplete.

**Fig. 4:**
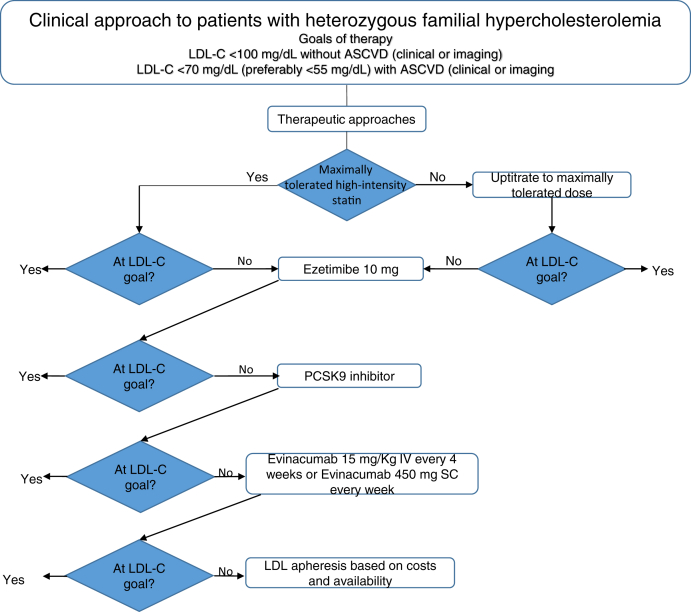
Clinical algorithm for LDL-cholesterol lowering in patients with heterozygous familial hypercholesterolemia. PCSK9, PCSK9, proprotein convertase subtilisin Kexin type 9; SC, subcutaneously.

Treatment with evinacumab reduced LDL-cholesterol by 50–56% in patients with elevated LDL-cholesterol levels on treatment with maximally tolerated statins with or without ezetimibe and a PCSK9 inhibitor. Thus, evinacumab would be considered for LDL-cholesterol lowering in patients who are refractory to treatment with available therapies (Fig. 4).

### Antisense oligonucleotide

In a phase 1 study, use of an *N*-acetyl galactosamine-conjugated antisense drug to *ANGPTL3* mRNA (vupanorsen) was associated with a dose-dependent reduction in LDL-cholesterol that ranged from −1.3% to −32.9% (69). In a double-blind dose ranging study that included 105 patients with hypertriglyceridemia (≥150 mg/dl), diabetes, and hepatic steatosis, there were no significant changes in LDL-cholesterol with vupanorsen (74). These data suggest that the mechanism of ANGPTL3 inhibition is an important consideration for LDL-cholesterol efficacy.

A clinical algorithm for the use of evinacumab to lower LDL-cholesterol in patients with HoFH and HeFH is illustrated in Figs. 3 and 4. This paradigm is adapted from an algorithm proposed by the European Society of Cardiology/European Atherosclerosis Society consensus statement on the use of PCSK9 inhibitors (75) and extends the author's recommendation for incorporation of evinacumab based on interpretation of phase 2 and phase 3 clinical trial data. For patients with HoFH, the larger and more consistent LDL-cholesterol-lowering efficacy of evinacumab warrants consideration of an ANGPTL3 inhibitor before initiation of a PCSK9 inhibitor, particularly in patients with null-null mutations or homozygous for *LDLR*, *LDLRAP1*, or *PCSK9* (47). The basis for this recommendation is the reduced efficacy of PCSK9 inhibition in lowering LDL-cholesterol lowering in these HoFH who were enrolled in the Efficacy and Safety of Evinacumab in Patients With Homozygous Familial Hypercholesterolemia trial. Alternatively, evinacumab should replace a PCSK9 inhibitor in patients with either HoFH or HeFH who have had a negligible response to a PCSK9 inhibitor or initiated in patients with HoFH who respond to a PCSK9 inhibitor. Future studies with evinacumab should consider the efficacy of this treatment on facilitating regression of tendon xanthomata with magnetic resonance imaging and coronary atherosclerosis with computerized tomography angiography (76).

## Inhibitors of Lp(a)

Selective agents that inhibit Lp(a) have entered clinical testing in patients with high levels of Lp(a) with and without FH. In a phase 2 trial, APO(a)-L_Rx_ (pelicarsen), an antisense oligonucleotide, was investigated in 286 patients with established cardiovascular disease and Lp(a) levels ≥150 nmol/l (77). The study included 27% with FH. Treatment with pelicarsen (subcutaneous administration every 4 weeks) resulted in dose-dependent reductions in Lp(a) from 23 to 81% with doses of 20–60 mg daily. HORIZON is a phase 3 clinical outcomes trial in patients with prior myocardial infarction, stroke, or revascularization for lower extremity arterial disease with Lp(a) concentrations >175 nmol/l (*ClinicalTrials.gov* identifier: NCT04023552). Eligible patients will be randomized to subcutaneous administration of pelicarsen 80 mg or matching placebo every 4 weeks. A small mRNA inhibitor to Lp(a) is under evaluation in a phase 2 dose-ranging trial in patients with ASCVD with Lp(a) levels ≥150 nmol/l (*ClinicalTrials.gov* identifier: NCT04270760). Eligible patients will be randomized to subcutaneous administration of olipasiran or matching placebo every 12 weeks. Pending the results from trials of cardiovascular outcomes, selective inhibition of Lp(a) would be considered adjunctive therapy in FH.

## Conclusions

FH is a common monogenic disorder of LDL metabolism associated with early onset ASCVD. Multiple classes of LDL-cholesterol-lowering therapies with proven clinical efficacy in lowering the risk of ASCVD events are conventionally prescribed in combination. However, most patients with HoFH and fewer patients with refractory HeFH, including those taking a maximally tolerated high-intensity statin, an ezetimibe, and a PCSK9 inhibitor, have persistent elevations in LDL-cholesterol requiring the use of expensive and poorly tolerated medications or apheresis procedures. In these difficult-to-treat patients, including HoFH patients with null-null traits in *LDLR*, evinacumab lowers LDL-cholesterol levels by about 50%. Thus, the future availability of a therapy that lowers LDL by an *LDLR*-independent mechanism allows for more patients with FH to achieve minimal acceptable LDL-cholesterol goals and potentially alter the trajectory of atherosclerotic cardiovascular events. The future availability of selective inhibitors of Lp(a) may provide a complementary approach to lowering the risk of atherosclerotic cardiovascular events pending determination of a reduction in cardiovascular events.

## Conflict of interest

R. S. R. has received grants from Amgen, National Institutes of Health, Novartis, and Regeneron. R. S. R. is on the advisory boards of Amgen, Amyrt, C5, CVS Caremark, Novartis, Regeneron, and 89 Bio. R. S. R. has received honoraria for nonpromotional speaking from Amgen, Kowa, and Regeneron. R. S. R. has stock holdings from MediMergent, LLC and royalties from Wolters Kluer (UpToDate). The content is solely the responsibility of the authors and does not necessarily represent the official views of the National Institutes of Health.
